# Effect of Various OR Noise on Fine Motor Skills, Cognition, and Mood

**DOI:** 10.1155/2019/5372174

**Published:** 2019-07-04

**Authors:** Cara Marie Rogers, Hannah Palmerton, Brian Saway, Devin Tomlinson, Gary Simonds

**Affiliations:** Virginia Tech Carilion School of Medicine and Research Institute, 2 Riverside Circle, Roanoke, VA 24016, USA

## Abstract

**Background:**

The amalgam of noises inherent to the modern-day operating room has the potential of diluting surgeon concentration, which could affect surgeon performance and mood and have implications on quality of care and surgeon resilience.

**Objective:**

Evaluate the impact of operating room environmental noises on surgeon performance including fine motor dexterity, cognition, and mood.

**Methods:**

37 subjects were tested under three different environmental noise conditions including silence, a prerecorded soundtrack of a loud bustling operating room, and with background music of their choosing. We used the Motor Performance Series to test motor dexterity, neuropsychological tests to evaluate cognitive thinking, and Profile of Mood States to test mental well-being.

**Results:**

Our results showed that typical operating room noise had no impact on motor dexterity but music improved the speed and precision of movements and information processing skills. Neurocognitive testing showed a significant decrement from operating room noise on verbal learning and delayed memory, whereas music improved complex attention and mental flexibility. The Profile of Mood States found that music resulted in a significant decrease in feelings of anger, confusion, fatigue, and tension along with decreased total mood disturbance, which is a measure of psychological distress. Loud operating room noise had a negative impact on feelings of vigor but no increase in total mood disturbance.

**Conclusion:**

Our results suggest that loud and unnecessary environmental noises can be distracting to a surgeon, so every effort should be taken to minimize these. Music of the surgeons' choosing does not negatively affect fine motor dexterity or cognition and has an overall positive impact on mood and can therefore be safely practiced if desired.

## 1. Introduction

All surgeons are familiar with the near constant din of the modern-day operating room. Ambient noise can become raucous with the myriad of intrinsic and extrinsic noises inherent to the typical operating room. Intrinsic noises include necessary conversations, alarms, surgical device noise including suction and cautery machines, shift changes, and surgical case counts. Extrinsic sources of noise include unnecessary conversations, phones and beepers, computers, doors slamming, traffic throughout the room, and hallway noise. Some surgeons even choose to alter the environmental noise in the operating room with background music. This amalgam of noises has the potential of diluting surgeon concentration from the task at hand, which could affect patient care. The relentless daily clamor could also influence surgeon mood, which could mount over a career and impact resilience and contribute to burnout. It is imperative to identify sources of potential surgeon distraction and mitigate these risks to patient safety and physician resilience. Therefore, we sought to investigate the impact of operating room environmental noises on surgeon performance including fine motor dexterity, cognition, and mood, as this could have implications on quality of care and surgeon resilience.

## 2. Methods

### 2.1. Instruments

We utilized the MLS Motor Performance Series of the Vienna Test Series by Schuhfried to assess fine motor dexterity. This is a modular test that utilizes Edwin Fleishman's factor analysis of manual dexterity. It consists of a panel with various contact surfaces, and the subject uses a stylus to perform static and dynamic tasks. We tested each subject's dominant and nondominant arm in the Steadiness, Aiming, Tapping, and Line Tracking tasks. These tests measure the accuracy and precision of movement, steadiness, finger dexterity, and speed of finger, wrist, and arm movements [1, 2].

Cognitive thinking skills were evaluated using a battery of standardized paper-pencil neuropsychological tests that have undergone extensive validation studies. The battery consisted of Hopkins Verbal Learning Test-Revised (Johns Hopkins University, Baltimore, Maryland), Brief Visuospatial Memory Test-Revised (Psychological Assessment Resources, Inc., Lutz, Florida), Trail Making Test Form B (Reitan Neuropsychological Laboratory, Tucson, Arizona), Symbol Digit Modalities Test (Western Psychological Services, Los Angeles, California), and the Stroop Test (Stoelting Company, Wood Dale, Illinois). Hopkins Verbal Learning Test was used to measure verbal learning and memory [3, 4]. Brief Visuospatial Memory Test was used to assess visuospatial memory [3, 5, 6]. Trail Making Test was utilized to evaluate the speed of processing, mental flexibility, and executive function [7, 8]. Symbol Digit Modalities Test gauged attention, visual scanning, tracking, and motor speed [9–11]. The Stroop Test measured selective attention, cognitive flexibility, and processing speed [12–14]. Clinical validity and reliability have been well established for this battery of tests in the evaluation of cognitive thinking abilities.

To evaluate for the effect on mental well-being, we utilized the Profile of Mood States created by McNair et al. in 1981. This is a self-reported inventory to assess transient and enduring mood changes. It consists of 65 items that describe emotional states, and the subjects grade each on a five-point Likert scale. The items are grouped into emotional states of anger and hostility, confusion and bewilderment, depression and dejection, tension and anxiety, vigor and activity, fatigue and inertia, and friendliness. This is a commonly used tool used by psychologists to measure psychological distress [15].

### 2.2. Testing

We tested 37 subjects comprised of 10 undergraduate students, 22 medical students, and 5 neurosurgical residents. Each subject was tested in randomized order under three different environmental noise conditions: silence, a loud operating room, and background music of their choosing. Silence was utilized as a control for baseline performance and compared to the two other environmental sound conditions which were administered in alternating order to participants in order to randomize conditions. Subjects were tested while listening to a prerecorded soundtrack of a typical modern-day operating room and while listening to music of their own choosing. The subjects' fine motor dexterity, cognitive thinking abilities, and mood state were tested under each condition. We then analyzed the data for statistically significant changes in the cohort performance under each condition. The average age of subjects was 29 years of age. There were 45% female and 55% male subjects. This study was conducted under the review of and with the prior approval of the Carilion Clinic Institutional Review Board and consent of the participants.

### 2.3. Statistical Analysis

All participant responses were pooled and analyzed as a single group as there were not enough participants in each subgroup of medical students, residents, and undergraduate students to offer a statistically significant result. The scores for each subtest were reported numerically and statistically analyzed to assess for any significant effects on performance. Paired sample *t*-tests were used to assess for statistically significant differences in performances between silence and operating room noise and between silence and music of the subject's choosing. The cutoff value for statistical significance was chosen to be 0.05 to ensure at least a 95% confidence interval in the results.

## 3. Results

### 3.1. MLS Motor Performance Series

The steadiness subtest measured a subject's ability to position the arm and hand precisely and hold this position for a prolonged period [1, 2]. It required subjects to insert the 2 mm tip of the stylus into a 5 mm hole on the platform and maintain this position for 32 seconds. Each time that the stylus touched the sides or bottom of the panel, it was recorded as an error. Performance was measured as the number of errors. There was no difference in errors between silence and operating room noise for either dominant (*p*=0.253) or nondominant arm (*p*=0.092) (see Table 1). There was also no difference in errors between silence and music of the subjects choosing for either dominant (*p*=0.079) or nondominant arm (*p*=0.058). Statistically significant results are displayed in Table 1.

The aiming task is a measure of hand-eye coordination and precision of movements [1, 2]. It required the subject to tap a line of twenty 5 mm diameter copper discs lined 4 mm apart, in succession with the stylus as quickly and precisely as possible. Each time that the stylus touched outside of the copper discs, it was recorded as an error. Performance was measured as the number of errors and by the amount of time it took the subject to complete the task. There was no difference in errors between silence and operating room noise for either dominant (*p*=0.186) or nondominant arm (*p*=0.055). There was also no difference in errors between silence and music of the subjects choosing for either dominant (*p*=0.446) or nondominant arm (*p*=0.057). There was no difference in the time it took to complete the task between silence and operating room noise for either dominant (*p*=0.057) or nondominant arm (*p*=0.475). There was a decrease in the amount of time to complete the task with music for both dominant (*p*=0.003) and nondominant arm (*p*=0.047).

The tapping subtest measures wrist-finger speed and the speed of untargeted movements [1, 2]. The subject is instructed to tap the stylus against a square metal plate as rapidly as possible for 32 seconds. Performance was measured as the number of taps recorded by the panel in this time period. There was no difference in performance with operating room noise for the dominant (*p*=0.353) or nondominant arm (*p*=0.29). Music resulted in an improved speed of tapping with the nondominant arm (*p*=0.038), but no change with the dominant arm (*p*=0.345).

The line tracking subtest evaluated the precision of arm-hand movements and information processing [1, 2]. This task required the subject to insert the stylus into a channeled maze that was 5 mm in width and proceed through it without touching the sides or bottom of the panel. Each time that the stylus touched the sides or bottom of the panel, it was recorded as an error. Performance was measured by the number of errors and the time it took to complete the maze. There was no significant difference in error rate of the dominant arm (*p*=0.168) or nondominant arm (*p*=0.083) with operating room noise but there was a significant decrement in speed of both the dominant arm (*p*=0.036) and nondominant arm (*p*=0.004). Music of the subjects' choosing resulted in a decreased error rate of the dominant arm (*p*=0.043) and no change in the nondominant arm performance (*p*=0.478). There was no change in the speed of either arm with music (*p*=0.48, *p*=0.087).

### 3.2. Paper-Pencil Neuropsychological Tests

The Hopkins Verbal Learning Test is a measure of verbal learning, immediate memory, and delayed memory [3, 4]. Subjects were read a list of twelve words consisting of four words from three semantic categories. They were instructed to immediately recall as many of the words as they could in any order. This immediate recall test was repeated three times as trial 1–3. Trial 4 was completed after the other neuropsychological tests in which the subject was instructed to recall as many of the words from the original list to assess delayed memory. Subjects were given one point for each word recalled during the immediate and delayed recall trials. Immediate recall ability was measured as the sum of trials 1, 2, and 3. Retention ability was measured by the higher of trial 2 or 3 minus trial 4 multiplied by 100. There was a decrement in immediate recall (*p*=0.015) and retention (*p*=0.02) with operating room noise. Music had no significant impact on either immediate recall (*p*=0.112) or retention (*p*=0.213).

The Brief Visuospatial Memory Test is a measure of visuospatial memory [3, 5, 6]. It consists of a page of six simple figures arranged in two columns and three rows. The subject is allowed to study the page for ten seconds and is then instructed to draw the figures on a blank sheet of paper. They are given one point for each figure they draw correctly and another point if the location on the page is correct for a maximum score of twelve. There was no statistically significant difference in performance with operating room noise (*p*=0.162) or music of the subjects' choosing (*p*=0.164).

The Trail Making Test is a measure of complex attention, mental flexibility, and visual-motor speed [7, 8]. The subjects are given a sheet of paper with 25 randomly arranged circles containing the numbers 1–13 and the letters A-L. They are given a pen and instructed to connect numbers to letters in ascending order. Performance is measured by the speed with which they complete the task. Operating room noise had no impact (*p*=0.49) yet music resulted in improved performance (*p*=0.03).

The Symbol Digit Modality Test is a measure of psychomotor speed, short-term memory attention, and concentration [9–11]. It involves a simple substitution task in which subjects are given a reference key and required to pair numbers with their respective geometric figures. Performance was graded by the number of geometric figures they are able to convert into numbers in 90 seconds. There was no appreciable difference in performance on this test with either operating room noise (*p*=0.215) or music (*p*=0.175).

The Stroop Test is a test of cognitive flexibility and response inhibition [12–14]. Subjects are given a sheet of paper with five columns of twenty words. The words consist of the red, green, and blue but the written color is different than the printed color. The subjects are instructed to read out loud the printed colors and not the written words. Their performance is measured as the number of words they read out loud correctly in 45 seconds. Operating room noise (*p*=0.154) and music (*p*=0.27) had no significant impact on performance for this test.

### 3.3. Profile of Mood States

The Profile of Mood States was used to evaluate for changes in mood or psychological distress in response to the environmental noise conditions that the subjects were exposed to [15]. The subjects were given a list of 65 words or statements that described feelings and asked to grade each based on how they felt. They used a 5-point Likert scale ranging from 0 for feelings they had not experienced to 5 for extreme feelings. The scores were grouped into mood categories including: anger and hostility, confusion and bewilderment, depression and dejection, tension and anxiety, vigor and activity, fatigue and inertia, and friendliness.

In comparison to silence, operating room noise resulted in no significant change in feelings of anger and hostility (*p*=0.312), confusion and bewilderment (*p*=0.404), depression and dejection (*p*=0.181), fatigue and inertia (*p*=0.121), or tension and anxiety (*p*=0.441). Operating room noise did decrease feelings of vigor and activity (*p*=0.044). Music of the subjects' choosing resulted in a significant decrease in feelings of anger and hostility (*p*=0.005), confusion and bewilderment (*p*=0.004), fatigue and inertia (*p*=0.005), and tension and anxiety (*p*=0.032). There were no changes in feelings of depression and dejection (*p*=0.064) or vigor and activity (*p*=0.382) with music. Total mood disturbance is calculated by adding the scores for tension, depression, anger, fatigue, and confusion and then subtracting the score for vigor. There was no significant change in the total mood disturbance with operating room noise (*p*=0.31) but there was an improvement in total mood disturbance with music of the subjects choosing (*p*=0.001).

### 3.4. Comparison between Students and Residents

We then tried to understand if the education and experience level of the participants impacted the motor and cognitive skills in different sound environment. For example, does a neurosurgical resident gain more improvement when listening to music versus OR noise compared to the improvement of an undergraduate student when listening to music versus OR noise. For the tests with statistical significance, we performed an unpaired, two-tail Student's *t*-test on the task improvement for each given participant in different environments between the residents (*n*=5) and the medical student/undergraduate (*n*=32) samples. There was no statistically significant change in task improvement at the 0.05 confidence level found between the two education level groups.

## 4. Discussion

Operating room noise can affect a surgeon's concentration, ability to discriminate speech, and memory [16]. Surgeons are more distracted when noise levels are higher [17]. To combat these necessary sounds in the operating room, some surgeons choose to play music. There are myriad reasons that surgeons would want or would not want music playing during surgery. The literature is inconclusive; there are studies that support both the inclusion and exclusion of music in the operating room. Some surgeons believe that the addition of extraneous, unnecessary sound adds even more stimuli to an already hectic environment. Other surgeons believe that music helps negate the loud and harsh sounds of the operating room, allowing them to focus and perform better. Allowing the participant to choose the music playing when completing the fine motor dexterity, cognition, and mood tasks is a novel metric that has not been analyzed before.

The present study demonstrated that operating room noise resulted in poorer performance on the Line Tracking Test, the Hopkins Verbal Learning Test, and the Profile of Mood. These scores can be translated to decrements in participants' arm-hand movement, information processing, immediate recall, retention, and decreases in feelings of vigor and activity. Music, however, resulted in improvements on the Aiming Task, Tapping Task, Line Tracking Test, Trail Making Test, and Profile of Mood. This correlates to improvements in hand-eye coordination, precision of movements, wrist-finger speed, speed of untargeted movements, arm-hand movement, information processing, complex attention, mental flexibility, visual-motor speed, and mood disturbance. Decreases in anger and hostility, confusion and bewilderment, fatigue and inertia, and tension and anxiety were also observed with music. The Hopkins Verbal Learning Test was not affected by music.

The results from the present study indicate that the addition of music to the operating room can be beneficial to a surgeon's ability and mood. Music is known to have anxiolytic effects, and surgeons reported fewer feelings of anger, hostility, fatigue and anxiety with the addition of music of their choosing to the tasks presented. Better mood states may lead to better surgical performance and therefore better patient care and may lead to decreased rates of burnout [18]. Better mood states may also improve and lengthen surgeons' careers.

Limitations of this study include a small sample size composed of undergraduate students, medical students, and neurosurgical residents. Furthermore, the majority of our sample was not experienced in surgery. The present study required participants to complete tasks outside of the operating room with prerecorded operating room noise being played. For the few participants with operating room experience, this setting may not have simulated the state of an operating room that they would be conditioned to. Future directions of work of this nature may consider including the variables of music and operating room noise, operating room noise with white noise, and music not of the participants' choosing. The effects of music may vary if the music is not chosen by the participant; this is a common occurrence in operating rooms, as the lead surgeon would be the one to decide the music. Different types of music may be relaxing to one individual, but stressful to another. Data should also be collected from participants working on a virtual surgery or a more mentally taxing set of tasks.

## 5. Conclusion

The inconclusive literature and varying opinions on music in the operating room has initiated a debate among surgeons. Through this study, we suggest that excessive and unnecessary noise can be distracting to surgeons and limit their ability to perform well. We conclude that operating room noise leads to decreased performance on tasks associated with fine motor dexterity and cognition and is disadvantageous to mood. We also conclude that listening to music is not detrimental to a surgeon's ability to perform tasks associated with fine motor dexterity and cognition and that music is beneficial to a surgeon's mood. Music, therefore, can be a positive addition to an operating room and can be beneficial to the surgeon and operating team if implemented safely and considerably.

## Figures and Tables

**Table 1:** Significant differences in tests of fine motor skills, cognition, and mood in the various noise conditions.

	OR noise vs. silence	Music vs. silence
Fine motor skills	(i) Decreased time to completion of line tracking task in both dominant (*p*=0.036) and nondominant hands (*p*=0.004)	(i) Increased tapping speed of nondominant hand (*p*=0.038) (ii) Decreased time to completion of aiming task in dominant (*p*=0.003) and nondominant hands (*p*=0.047) (iii) Decreased error rate in line tracking test in dominant hand (*p*=0.043)
Cognition	(i) Decreased immediate (*p*=0.015) and delayed recall (*p*=0.002)	(i) Improved performance on test of complex attention, mental flexibility, and visual-motor speed (*p*=0.03)
Mood	(i) Decreased feelings of vigor and activity (*p*=0.044)	(i) Decreased feelings of anger and hostility (*p*=0.005), confusion and bewilderment (*p*=0.004), fatigue and inertia (*p*=0.005), and tension and anxiety (*p*=0.032) (ii) Improved total mood disturbance (*p*=0.001)

## Data Availability

The data used to support the findings of this study are available from the corresponding author upon request.
